# Investigation of multiple nosocomial infections using a semi-Markov multi-state model

**DOI:** 10.1186/s13756-024-01421-5

**Published:** 2024-06-06

**Authors:** Xiao Zhong, Dong-Li Wang, Li-Hua Xiao, Yan Liu, Shan-Wen Yang, Lan-Fang Mo, Qin-Fei Wu, Mei Lin, Lan-Fang He, Xiao-Feng Luo

**Affiliations:** 1 Infection Management Department, Shenzhen Guangming District People’s Hospital, Shenzhen, Guangdong 518106 China; 2 Testing Centre, Guangming District Centre for Disease Control and Prevention, Shenzhen, Guangdong China

**Keywords:** Nosocomial infection, Multiple nosocomial infection, Semi-Markov multi-state model, Cumulative risk, Risk factors, Length of stay

## Abstract

**Background:**

The prevalence of multiple nosocomial infections (MNIs) is on the rise, however, there remains a limited comprehension regarding the associated risk factors, cumulative risk, probability of occurrence, and impact on length of stay (LOS).

**Method:**

This multicenter study includes all hospitalized patients from 2020 to July 2023 in two sub-hospitals of a tertiary hospital in Guangming District, Shenzhen. The semi-Markov multi-state model (MSM) was utilized to analyze risk factors and cumulative risk of MNI, predict its occurrence probability, and calculate the extra LOS of nosocomial infection (NI).

**Results:**

The risk factors for MNI include age, community infection at admission, surgery, and combined use of antibiotics. However, the cumulative risk of MNI is lower than that of single nosocomial infection (SNI). MNI is most likely to occur within 14 days after admission. Additionally, SNI prolongs LOS by an average of 7.48 days (95% Confidence Interval, CI: 6.06–8.68 days), while MNI prolongs LOS by an average of 15.94 days (95% CI: 14.03–18.17 days). Furthermore, the more sites of infection there are, the longer the extra LOS will be.

**Conclusion:**

The longer LOS and increased treatment difficulty of MNI result in a heavier disease burden for patients, necessitating targeted prevention and control measures.

**Supplementary Information:**

The online version contains supplementary material available at 10.1186/s13756-024-01421-5.

## Backgroud

A nosocomial infection, acquired by a patient during their hospitalization [1], is associated with a substantial disease burden [2]. The reported incidence of NIs varies significantly across regions, with rates of 3.2% in the United States and 6.5% in the European Union. However, the worldwide prevalence is likely much higher [3, 4]. According to the European Center for Disease Control and Prevention's reports [5], within Europe, approximately 81,000 individuals acquire NIs daily, resulting in around 150,000 annual fatalities and an extended LOS totaling 16 million days. NIs not only compromise patient health but also impose significant economic burdens.

The occurrence and progression of NIs often lead to the simultaneous infection of multiple organs, tissues, or sites, termed MNIs [6]. MNIs are notably prevalent, representing 10.3% of all NIs [7]. In Intensive Care Units (ICUs), NIs are particularly concerning, affecting approximately 71% of patients and resulting in a significantly prolonged LOS compared to single infections—57.9 versus 30.0 days, respectively [6]. A study among ICU patients revealed a higher prevalence of MNIs among males, who were 5 to 12 years older on average and experienced an extended LOS of 13 to 28 days compared to those with single NIs [8]. However, these studies are relatively outdated and predominantly descriptive. There is a significant geographical variation in the prevalence of MNIs across different regions in China, with rates ranging from 4.31% to 11.59% reported in tertiary hospitals [9–14]. Importantly, the lack of systematic studies using a multi-state model to analyze MNIs in China is evident, underscoring the need for more advanced analytical approaches.

In addressing the research gap concerning the progression of MNIs, our study employed a semi-Markov MSM [15] to conduct a systematic investigation. This advanced model, which accounts for the time spent in each health state, offers a more precise prediction of transition probabilities compared to traditional MSMs. It also enables a comprehensive assessment of risk factors and LOS in specific disease states. The semi-Markov MSM is particularly adept at addressing time-dependent biases and competitive risks, providing a sophisticated method for studying MNIs [16]. By incorporating transfer-specific risk factors, cumulative risk, and the probability of transfer, our investigation yielded a nuanced understanding of MNIs' natural course. This knowledge is essential for raising awareness and guiding the development of targeted prevention and control strategies.

Our study builds upon the seminal work of Stewart S et al. [17], who identified a critical 14-day period post-admission for the occurrence of most NIs. This timeframe is vital for implementing preventive strategies, as it presents a window of opportunity for interventions that could reduce the incidence of NIs. Additionally, we consider the findings of Habibollah et al. [2], who reported an 8.09 ± 0.91 days increase in LOS due to NIs, with CNS infections extending LOS by 24.42 days. These findings underscore the severe impact of CNS infections on patient care, highlighting the need for focused interventions.

Expanding on the work of Kritsotakis et al. [18], who found that MNIs extend LOS by 16.6 days, our study corroborates and extends this finding. We provide further evidence of the significant impact of MNIs on healthcare systems, noting that the extension in LOS affects patient outcomes and has broader implications for healthcare resource allocation and hospital planning.

The central research question our study addresses is: How does the semi-Markov MSM offer insights into MNIs' progression and inform the development of clinical strategies to mitigate their impact? Our analysis, leveraging the semi-Markov MSM, uncovers the complex dynamics of MNIs and identifies potential intervention points that could significantly improve patient outcomes. Our findings contribute to the ongoing discourse by stimulating further inquiry into the relationship between infection control, patient care, and NI prevention.

## Methods

### Research subject

**Fig. 1:**
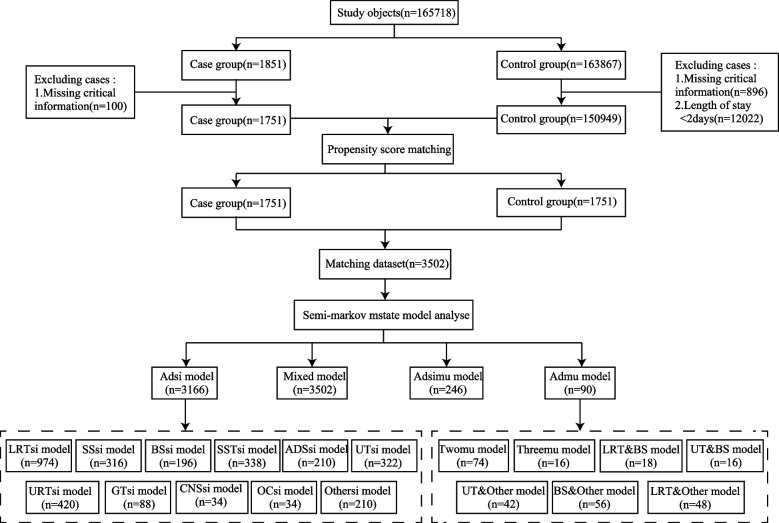
The study flow chart. Note. Single Nosocomial Infection, Si, Multiple Nosocomial Infection, Mu, Lower Respiratory Tract, LRT, Surgical Site, SS, Blood System, BS, Skin and Soft Tissue, SST, Abdomen and Digestive System, ADS, Urinary Tract, UT, Upper Respiratory Tract, URT, Genital Tract, GT, Central Nervous System, CNS, Oral Cavity, OC

The multicenter study was conducted in two sub-hospitals of a 1350-bed tertiary public hospital located in Guangming District, Shenzhen, China. One hospital comprised 900 beds situated in the western region of Guangming District, while the other hospital consisted of 450 beds located in the eastern region of Guangming District. The study encompassed all patients who were admitted to the hospital between January 2020 and July 2023. The case group comprised all patients who acquired NIs during the study period, while the control group consisted of patients without NIs during the same period. Patients with a LOS of less than 48 h in both groups, as well as those with missing demographic information, clinical details, DRG grouping, antimicrobial usage data, and invasive procedure records were excluded. The flowchart illustrating the screening process for research subjects is presented in Fig. 1.

### Diagnosis of NIs

The diagnosis of NIs was based on the Diagnostic Criteria for Nosocomial Infection 2001 (Trial) [19] issued by the Ministry of Health of the People's Republic of China (The English version can be found in Supplementary Material 1.). Upon the initial detection of a NI by clinical physicians, it is reported to the Infection Control Department through the Nosocomial Infection Surveillance Information System (NISIS). This is followed by an assessment conducted by two experienced infection control specialists who determine, based on established criteria, whether the infection is indeed nosocomial. In cases where the two specialists' opinions diverge, a third specialist is called upon to make the final decision. Furthermore, the NISIS is integrated with interfaces to the Hospital Information System (HIS), Laboratory Information System (LIS), Radiology Information System (RIS), Operating Room Management System (ORMS), and Medical Record Information System (MRIS). It provides early warnings for potential NIs by monitoring various patient indicators such as body temperature, bacterial detection, inflammatory markers, chest radiography findings, and the use of antimicrobial agents. The two aforementioned infection control specialists are then responsible for evaluating these alerts to confirm whether they represent actual NIs. MNIs refer to occurrences of NIs affecting more than one distinct anatomical site within a patient. These infections are not considered as a single entity due to their potential for diverse etiologies, severities, and implications on patient care. The microorganisms identified from different infection sites in patients may be either identical or diverse [6].

### Patient classification and data collection methodology

Diagnosis Related Groups (DRGs) [20] are pivotal in evaluating the quality and efficacy of medical services and are instrumental in the medical insurance reimbursement process. The DRG system classifies patients into diagnostic groups based on a variety of factors including age, disease diagnosis, comorbidities, complications, treatment modalities, disease severity, and resource utilization. This classification ensures that patients within the same DRG share similar or identical illness severity levels. Each month, the medical records management department uploads the data of all patients discharged in the previous month into the CN-DRG system. This process automates the determination of DRG categories and calculates the corresponding relative weight (RW) values for individual patients. Patients who do not meet the enrollment criteria for DRG categorization are designated as non-enrollment cases. Furthermore, the collection of indicator data is facilitated by the NIIS, which provides patient demographic information, NI data, microbial test results, and clinical details including diagnosis and treatment records. The DRGs for each patient are derived from the CN-DRG system, rounding out the dataset essential for our analysis.

### Research methodology and design

The case–control study design method was employed in this study. Initially, a propensity score matching (PSM) technique was utilized to effectively address the baseline confounding factors between the case groups and control groups. The semi-Markov MSM was subsequently employed to investigate the transfer-specific risk factors, cumulative risk, probability of transfer, and extended LOS associated with both SNIs and MNIs in the matched dataset. The retrospective nature of this study received approval from the hospital Ethics Committee, thereby obviating the need for informed consent from patients. To ensure patient privacy, certain individuals' names and hospitalization numbers were anonymized and replaced with unique identifiers. The research methodology is visually depicted in Fig. 1.

### Methodology for microbiological detection

The VITEK2 bacterial identification drug sensitivity analyzer (Biomerieux, Merieux Alliance, France) was employed for the identification of bacterial microorganisms. The microbiological identification protocol adheres to the guidelines set forth by the Clinical Laboratory Standards Institute (CLSI).

### The utilization of PSM technique

Logistic regression, a well-established statistical method for binary outcome prediction [21], was employed to calculate the propensity scores of patients in each group. This technique is fundamental in estimating the likelihood of treatment assignment based on observed covariates, thereby mitigating selection bias in observational research [22]. Adhering to the principles of nearest neighbor and caliper matching, we meticulously conducted a 1:1 case–control matching with a caliper width set at 0.2 to ensure group comparability. The covariates for matching were selected for their clinical significance and potential impact on outcomes, encompassing patient gender, age, admission with infection (AI), admission route (Emergency, Outpatient, Transferred from other medical institutions), Charlson comorbidity score [23], relative weight (RW), surgery (defined as procedures performed exclusively in the operating room), invasive mechanical ventilation (IMV), central venous catheterization (CVC) duration, and indwelling catheter (IC) presence.

### Data analysis of multi-state models

#### The establishment of multi-state model

**Fig. 2:**
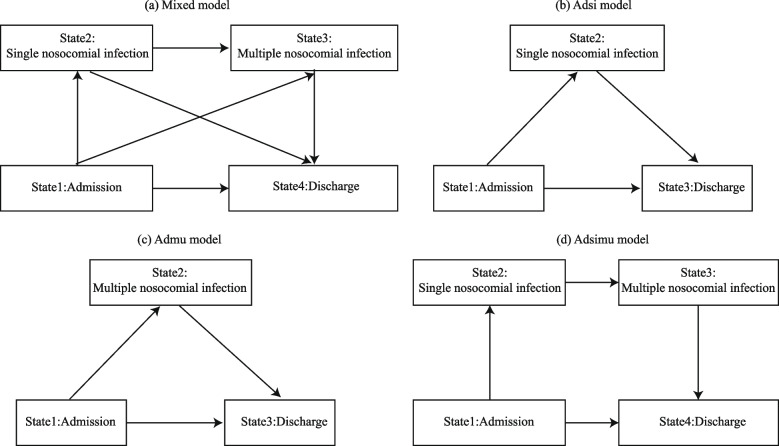
The multi-state models employed in this study. The diagram (**a**) is divided into four states, representing the natural history of nosocomial infection occurrence. Due to insufficient deaths, death and discharge statuses were combined, and the transfer routes were ignored due to few cases of multiple nosocomial infection transferring to single nosocomial infection. The diagrams (**b**) and (**c**) have only three states each for research purposes, with state 2 representing single or multiple nosocomial infections. The diagram (**d**) omits the paths of single nosocomial infection transfer to discharge and admission transfer to multiple nosocomial infection

In this study, we developed four distinct multi-state models to delineate the transition of patients through various health states in relation to NI:

Model 1 (Mixed Model): This framework categorizes patients into four stages aligned with their clinical trajectory from hospital admission to potential NI and discharge. State 1 indicates the pre-infection period post-admission, highlighting NI susceptibility. State 2 is designated for SNIs, while State 3 accounts for MNIs. State 4 is an absorbing state representing either discharge or death, with both outcomes combined due to the rarity of fatal NI cases. Transitions are possible from State 1 to States 2, 3, or 4, and from State 2 to States 3 or 4. State 3 leads exclusively to State 4, with the transition from MNIs back to SNIs omitted due to limited patient data (Fig. 2a).

Model 2 (Adsi model): Patients can be directly transferred from state 1 (admission) to state 3 (discharge), as well as to state 2 (SNI). Additionally, patients in state 2 can transition to the discharge state. Figure 2b displays the model's structure, showing the possible transitions from admission to SNI and then to discharge. To investigate the impact of different infection sites, this model was applied to various scenarios corresponding to specific infection sites, including lower respiratory tract (LRT), surgical site (SS), blood system (BS), skin and soft tissue (SST), abdomen and digestive system (ADS), urinary tract (UT), upper respiratory tract (URT), genital tract (GT), central nervous system (CNS), oral cavity (OC), and other locations.

Model 3 (Admu model): Patients admitted in state 1 can be directly transferred to either state 3 (discharge) or state 2 (MNI), while patients in state 2 can be transferred to the discharge state. Figure 2c illustrates the model, detailing the direct transitions from admission to MNI and then to discharge. The model was adapted to examine different combinations of MNIs, resulting in seven distinct scenarios based on various infection combinations.

In model 4 (Adsimu model), after admission at state 1, patients may transition directly to state 4 (discharge) or state 2 (SNI). Patients in state 2 can only progress to state 3 (MNI) and not directly to state 4. Eventually, patients in state 3 will be transferred to state 4, as shown in Fig. 2d. These models provide a comprehensive tool for analyzing the dynamics of NI and patient outcomes, facilitating a nuanced understanding of infection risks and transitions.

#### Test of the Markov assumption

To evaluate the Markov properties of transition states within the MSM, we applied the global and local test methods as proposed by Gustavo Soutinho [24]. The Markov assumption posits that transition probabilities to the subsequent state are contingent solely upon the current state, dispensing with the history of previous states. Utilizing the likelihood ratio test to calculate *P* values, we assessed deviations of the observed transition probabilities from those implied by the Markov assumption. A non-significant *P* value (*P* > 0.05) confirms the model's adherence to the Markov property, simplifying state transition predictions by focusing on the most recent state.

Given the test results, which indicated no perfect adherence to the Markov property across all transitions, we opted for the semi-Markov MSM for our analysis. This selection was underpinned by the semi-Markov model's capacity to accommodate diverse sojourn time distributions, thereby capturing the unique dynamics of state transitions evident in our NI data. Unlike the traditional Markov model, which assumes exponential holding times, the semi-Markov MSM provides a more sophisticated representation of state durations, essential for our dataset where the Markov assumption was not universally valid. Crucially, the semi-Markov model preserves the Markovian decision-making process for transitions [15], enabling a nuanced and accurate depiction of the NI state dynamics observed in our study.

#### Introduction to a semi-Markov MSM [15]

To construct a semi-Markov stochastic process, consider the time homogeneous Markov chain ${\left\{{J}_{n}\right\}}_{n\ge 0}$ as the basis, the state space is: $\left\{\text{1,2},\dots ,l\right\}$, the transition intensity from state *i* to state$j\;\left(i\neq j\right)$ is $P_{ij}=A\left(J_n=j\left|J_{n-1}=i\right.\right)$. We define:

$$\sum_{j\neq i}p_{ij}=\left\{\begin{array}{c}1\,\mathrm{for}\,\mathrm{non}-\mathrm{absorbing}\,i,\\0\,\mathrm{for}\,\mathrm{absorbing}\,i.\end{array}\right.$$

The increasing order of the jump time is represented by *T*_*0*_ = 0 < *T*_*1*_ < *T*_*2*_ < *T*_*3*_ < …, defining the number of transitions to time *t* as $N\left(t\right)=max\left\{n:{T}_{n}<t\right\}$, where *t* ≥ 0. The random process *X*(*t*): = *J*_*N(t)*_ is a semi-Markov process, where upon entering state *i*, the subsequent state *j* is determined by transition probability *p*_*ij*_, and the duration of transitioning from state *i* to *j* follows a random variable with cumulative distribution function *F*_*ij*_(*t*): *F*_*ij*_(*t*) = *P*(*τ*_*n*_ ≤ *t*|* J*_*n*−*1*_ = *i*, *J*_*n*_ = *j)*,* t* ≥ *0*, where *τ*_n_ = *T*_*n*_ − *T*_*n−1*_, Therefore, semi-Markov processes do not exhibit Markovian properties and thus cannot be considered as Markov processes. Furthermore, semi-Markov processes allow for arbitrary distributions of stay times in any state while preserving the Markov properties for embedded (discrete-time) Markov chains ${\left\{{j}_{n}\right\}}_{n}\ge 0$.

#### Data distribution selection

The flexible parameter MSM is employed for semi-Markov model analysis, enabling the selection of parameter distribution types such as Weibull, exponential, Gompertz, gamma, log-logistic, log-normal, generalized gamma (gengamma), original generalized gamma (gengama.orig), generalized F (genf), and original generalized F (genf.orig). To fit and optimize the parameter distribution, the Akaike information criterion (AIC) [25] was utilized to screen the data distribution parameters of the regression model. Subsequently, the distribution with the lowest AIC value was chosen to establish the parameter regression model.

#### Analysis of risk factors associated with transfers

In the analysis of risk factors associated with patient transfers, transfer-specific regression models were employed, incorporating variables for propensity score matching and the use of antimicrobial agents. The use of antimicrobial agents, which may include the use of a single antibiotic or a combination of antibiotics as part of the treatment protocol. Additionally, the combined use of antimicrobial agents, characterized by the simultaneous or sequential application of multiple drugs to treat infections, was also factored into the models. The models were designed to account for the duration of the effects of these variables.

#### Multi-state analysis of NIs impact

In our analysis of the impact of NIs on LOS, we employed a methodological triad to comprehensively assess the outcomes. Firstly, to estimate the additional LOS caused by NI, we adhered to the approach by Schulgen and Schumacher [26], which involves evaluating the expected difference in LOS during the infected state at a given time point. We applied the Bootstrapping method [27] to calculate the CI for the prolonged LOS, enhancing the reliability of our estimates. This process was further refined by conducting a stratified analysis based on the specific infection sites, which allowed for a more granular understanding of the extra LOS associated with NI.

Secondly, the computation of cumulative transfer risk was undertaken using the transition-specific risk function ${H}_{ij}(t)={\int }_{0}^{t}{q}_{ij}(u)du$ which represents the cumulative risk of transitioning from state *i* to state *j* over time *t*. In the context of our work, this function is crucial for understanding the progression of NIs, as it captures the accumulated risk of moving from a non-infected state to an infected state, or between different infected states. Stratified analysis was conducted based on different sites of infection to explore how the cumulative risk varies across various anatomical locations, providing a more nuanced understanding of the infection dynamics.

Lastly, we predicted the transfer specificity probability within the MSM by calculating the probability of patients occupying a particular state at a given time, conditional on their initial state. Utilizing the R language software, we simulated the state history for a large cohort, defaulting to 10,000 simulations, to derive transition probability matrices at specific time intervals post-infection. This stratified analysis elucidated the distinct transition patterns across different infection sites, contributing to a more precise prediction of transfer probabilities.

### Statistical analysis

The data were entered into Excel 365 to compile a comprehensive database. PSM was performed using the Matchit package of R software (version 4.2.1). For the analysis of the MSM, we employed the Flexsurv package. The etm package was utilized to calculate the incremental LOS attributed to NIs. Continuous variables were described by either the mean and standard deviation (for normally distributed data) or the median and interquartile range (for non-normally distributed data). Group differences in continuous variables were compared using the appropriate test based on the normality assumption: the independent samples *t*-test for normally distributed data or the Wilcoxon rank sum test for data that failed normality tests. Categorical variables were presented as constituent ratios and analyzed using the Chi-square test or Fisher's exact test, as appropriate. A significance level of *P* < 0.05 was considered statistically significant for all comparisons.

## Results

### Data processing and baseline of the study

From January 2020 to July 2023, a total of 165,718 patients were hospitalized, with 152,700 enrolled after applying inclusion and exclusion criteria. This included 1,751 patients with NIs and 150,949 without. PSM successfully paired all case group patients, resulting in a MSM analysis of 3,502 individuals. Most baseline characteristics were balanced post-matching (*P* < 0.05), except for a higher proportion of surgical patients in the control group. The use of antibiotics was not considered in the matching, leading to slightly higher usage in the case group. The most prevalent NI pathogens were *Escherichia coli, Klebsiella pneumoniae, Pseudomonas aeruginosa, Staphylococcus aureus*, and *Candida albicans*. The top infection sites in the case group were the lower respiratory tract, urinary tract, upper respiratory tract, skin and soft tissue, and surgical sites, highlighting the predominance of these areas for NI occurrence—see Table 1.

**Table 1:** Baseline characteristics and balance assessment of subjects after PSM

Variable	Case group(*n* = 1751)	Control group (*n* = 1751)	*P*-value
**Characteristic**			
Age (mean (SD))	47.51(23.41)	47.52(21.78)	0.983
AI (%)	398(22.70)	379(21.60)	0.464
Gender (%)	701(40.0)	694(39.60)	0.836
RW (mean (SD))	2.82(3.14)	2.75(3.03)	0.548
Admission (%)			0.353
Emergency	809(46.20)	799(45.60)	
Others	75(4.30)	60(3.40)	
Outpatient	867(49.50)	892(50.90)	
Charlson index (mean (SD))	3.17(2.99)	3.07(2.87)	0.347
Surgery (%)	870(49.70)	947(54.10)	0.01
IMV (%)	241(13.80)	223(12.70)	0.397
CVC (%)	508(29.00)	492(28.10)	0.575
IC (%)	928(53.00)	963(55.00)	0.249
Use.Antibiotics (%)	1661(94.90)	1129(64.50)	< 0.001
Coantibiotics (%)	1172(66.90)	411(23.50)	< 0.001
**Pathogenes**			< 0.001
*Escherichia coli* (%)	207(19.08%)	19(6.86%)	
*Klebsiella pneumoniae* (%)	136(12.53%)	51(18.41%)	
*Pseudomonas aeruginosa* (%)	105(9.68%)	25(9.03%)	
*Staphylococcus aureus* (%)	98(9.03%)	15(5.42%)	
*Candida albicans* (%)	67(6.18%)	27(9.75%)	
*Acinetobacter baumannii* (%)	66(6.08%)	11(3.97%)	
*Enterobacter cloacae* (%)	48(4.42%)	3(1.08%)	
*Enterococcus faecalis* (%)	37(3.41%)	3(1.08%)	
*Stenotrophomonas maltophilia* (%)	28(2.58%)	11(3.97%)	
*Staphylococcus epidermidis* (%)	25(2.30%)	5(1.81%)	
*Enteroaerogen* (%)	19(1.75%)	4(1.44%)	
*Serratia marcescens* (%)	13(1.20%)	1(0.36%)	
*Staphylococcus hominis* (%)	13(1.20%)	3(1.08%)	
*Streptococcus pneumoniae* (%)	13(1.20%)	6(2.17%)	
*Proteus mirabilis* (%)	12(1.11%)	3(1.08%)	
*Candida glabrata* (%)	12(1.11%)	3(1.08%)	
*Ureaplasma Urealyticum* (%)	11(1.01%)	3(1.08%)	
*Monilia tropicalis* (%)	10(0.92%)	6(2.17%)	
Others (%)	165(15.21%)	78(28.16%)	
**Infection sites**			
LRT(%)	589(30.36%)	\	
UT(%)	229(11.80%)	\	
URT(%)	225(11.60%)	\	
SST(%)	187(9.64%)	\	
SS(%)	182(9.38%)	\	
BS(%)	174(8.97%)	\	
ADS(%)	119(6.13%)	\	
Others(%)	116(5.98%)	\	
GT(%)	55(2.84%)	\	
CNS(%)	31(1.60%)	\	
OC(%)	19(0.98%)	\	
PC(%)	11(0.57%)	\	
Bone & Joint(%)	3(0.15%)	\	

*SD* Standard Deviation, *AI* Admission with Infection, *RW* Relative Weight, *IMV* Invasive Mechanical Ventilation, *CVC* Central Venous Catheterization, *IC* Indwelling Catheter, *Use.Antibiotics* Use of Antibiotics, *Coantibiotics* Combined Use of Antibiotics, *LRT* Lower Respiratory Tract, *SS* Surgical Site, *BS* Blood System, *SST* Skin and Soft Tissue, *ADS* Abdomen and Digestive System, *UT* Urinary Tract, *URT* Upper Respiratory Tract, *GT* Genital Tract, *CNS* Central Nervous System, *OC* Oral Cavity, *PC* Pleural Cavity

### Transfers-specific regression results

The unstratified transfer specific regression analysis revealed that Age, AI, Gender (male), Admission (Others), Surgery, CVC, and IC were significant risk factors for patients transitioning from admission to SNI. The transfer of patients from SNI to MNI is influenced by factors such as Age, AI, Combined use of antibiotics. Infection on admission and Surgery were risk factors for transferring patients from admission to MNI, while Antibiotics and Combined use of antibiotics promoted the transfer from infection state to discharge state (refer to Table 2).

**Table 2:** Transfer specific regression results (Unstratified)

Factors	Models	Trans	Distribution	HR	Low95%CI	Up95%CI
Age	Adsi model	Ad → Si	gengamma	1.006	1.003	1.008
	Mixed model	Si → Mu	gompertz	1.006	1.003	1.008
AI	Adsi model	Ad → Si	gengamma	1.293	1.180	1.417
	Mixed model	Ad → Si	gengamma	1.293	1.180	1.417
	Admu model	Ad → Mu	lnorm	2.043	1.100	3.795
	Adsimu model	Si → Mu	lnorm	2.079	1.220	3.542
	Adsimu model	Mu → Dis	genf	1.421	1.131	1.786
Gender (Female)	Adsi model	Ad → Si	gengamma	0.881	0.820	0.947
	Adsi model	Si → Dis	llogis	0.798	0.742	0.858
	Mixed model	Ad → Si	gengamma	0.881	0.820	0.947
	Mixed model	Si → Dis	llogis	0.795	0.739	0.855
Admission (Others)	Adsi model	Ad → Si	gengamma	1.257	1.032	1.531
	Mixed model	Ad → Si	gengamma	1.257	1.032	1.531
Admission (Outpatient)	Mixed model	Si → Dis	llogis	0.850	0.788	0.916
	Adsi model	Si → Dis	llogis	0.865	0.802	0.932
Surgery	Adsi model	Ad → Si	gengamma	1.228	1.131	1.333
	Mixed model	Ad → Si	gengamma	1.228	1.131	1.333
	Mixed model	Ad → Mu	lnorm	2.303	1.237	4.286
	Mixed model	Ad → Mu	lnorm	2.303	1.237	4.286
IMV	Adsi model	Si → Dis	llogis	0.796	0.673	0.941
	Mixed model	Si → Dis	llogis	0.818	0.694	0.964
CVC	Adsi model	Ad → Si	gengamma	1.316	1.180	1.468
	Mixed model	Ad → Si	gengamma	1.316	1.180	1.468
IC	Adsi model	Ad → Si	gengamma	1.14	1.04	1.24
	Mixed model	Ad → Si	gengamma	1.14	1.04	1.24
Use.Antibiotics	Adsi model	Si → Dis	llogis	1.45	1.23	1.71
	Mixed model	Si → Dis	llogis	1.44	1.22	1.70
	Mixed model	Mu → Dis	llogis	3.71	1.14	12.05
	Adsimu model	Mu → Dis	genf	11.88	5.37	26.28
Coantibiotics	Adsi model	Si → Dis	llogis	1.34	1.23	1.45
	Mixed model	Si → Mu	gompertz	4.57	1.98	10.56
	Mixed model	Si → Dis	llogis	1.37	1.26	1.49
	Mixed model	Mu → Dis	llogis	2.67	1.40	5.10
	Admu model	Mu → Dis	llogis	3.29	1.39	7.79

*HR* Hazard Ratio, *CI* Confidence Interval, *AI* Admission with Infection, *RW* Relative Weight, *IMV* Invasive Mechanical Ventilation, *CVC* Central Venous Catheterization, *IC* Indwelling Catheter, *Use.Antibiotics* Use of Antibiotics, *Coantibiotics* Combined Use of Antibiotics, *Ad* Admission, *Si* Single Nosocomial Infection, *Mu* Multiple Nosocomial Infection, *Dis* Discharge

The regression results for transfers, stratified by infection site, identified Age, AI, RW, Admission (Outpatient), Charlson index, Surgery, CVC and IC as risk factors for transferring from admission to SNI. Use of antibiotics and Combined use of antibiotics were found to promote transfer from NI to discharge (see Table 3).

**Table 3:** Transfer specific regression results based on infection sites

Factors	Models	Trans	Distribution	HR	Low95%CI	Up95%CI
Age	ADS Adsi model	Ad → si	lnorm	1.014	1.007	1.022
	URT Adsi model	Ad → si	gengamma	1.010	1.000	1.010
	OC Adsi model	Si → Dis	gompertz	0.760	0.654	0.882
AI	LRT Adsi model	Ad → si	gengamma	1.506	1.277	1.776
	SST Adsi model	Ad → si	lnorm	1.418	1.060	1.898
	ADS Adsi model	Ad → si	lnorm	1.384	1.002	1.911
	Others Adsi model	Ad → si	gengamma	1.838	1.361	2.482
Gender	LRT Adsi model	Si → Dis	llogis	0.821	0.708	0.953
	URT Adsi model	Si → Dis	llogis	0.675	0.562	0.809
	SS Adsi model	Si → Dis	lnorm	0.778	0.607	0.999
	BS Adsi model	Ad → si	lnorm	0.703	0.522	0.946
	BS Adsi model	Si → Dis	weibull	0.677	0.556	0.824
	UT Adsi model	Ad → si	lnorm	0.687	0.544	0.868
	ADS Adsi model	Ad → si	lnorm	0.679	0.533	0.865
	GT Adsi model	Ad → si	gompertz	33.700	2.200	515.000
RW	SST Adsi model	Ad → si	lnorm	1.231	1.137	1.333
	ADS Adsi model	Ad → si	lnorm	1.090	1.001	1.187
	OC Adsi model	Si → Dis	gompertz	0.010	0.001	0.146
	URT Adsi model	Ad → si	gengamma	1.080	1.020	1.150
Admission (Outpatient)	LRT Adsi model	Ad → si	gengamma	1.162	1.009	1.338
	LRT Adsi model	Si → Dis	llogis	0.824	0.709	0.958
Charlson index	SST Adsi model	Ad → si	lnorm	1.082	1.005	1.165
	SS Adsi model	Si → Dis	lnorm	0.907	0.826	0.997
	UT Adsi model	Si → Dis	llogis	0.926	0.875	0.981
	OC Adsi model	Ad → si	llogis	1.410	1.147	1.732
Surgery	LRT Adsi model	Ad → si	gengamma	1.321	1.124	1.554
	UT Adsi model	Ad → si	lnorm	1.501	1.143	1.971
	OC Adsi model	Ad → si	llogis	3.647	1.776	7.490
	Others Adsi model	Ad → si	gengamma	1.473	1.120	1.937
CVC	SST Adsi model	Ad → si	lnorm	1.637	1.123	2.386
	UT Adsi model	Ad → si	lnorm	2.145	1.461	3.151
IC	SST Adsi model	Ad → si	lnorm	1.30	1.05	1.62
Use.Antibiotics	Others Adsi model	Si → Dis	weibull	1.58	1.00	2.49
Coantibiotics	LRT Adsi model	Si → Dis	llogis	1.27	1.08	1.50
	URT Adsi model	Si → Dis	llogis	1.27	1.05	1.54
	SST Adsi model	Si → Dis	lnorm	1.33	1.05	1.67
	UT Adsi model	Si → Dis	llogis	1.49	1.17	1.89
	ADS Adsi model	Si → Dis	genf	1.57	1.22	2.01
	GT Adsi model	Si → Dis	gompertz	0.13	0.04	0.42
	Others Adsi model	Si → Dis	weibull	1.39	1.04	1.84

*HR* Hazard Ratio, *CI* Confidence Interval, *LRT* Lower Respiratory Tract, *SS* Surgical Site, *BS* Blood System, *SST* Skin and Soft Tissue, *ADS* Abdomen and Digestive System, *UT* Urinary Tract, *URT* Upper Respiratory Tract, *GT* Genital Tract, *CNS* Central Nervous System, *OC* Oral Cavity, *AI* Admission with Infection, *RW* Relative Weight, *CVC* Central Venous Catheterization, *IC* Indwelling Catheter, *Use.Antibiotics* Use of Antibiotics, *Coantibiotics* Combined Use of Antibiotics, *Ad* Admission, *Si* Single Nosocomial Infection, *Mu* Multiple Nosocomial Infection, *Dis* Discharge

### Cumulative cause-specific hazards in MSM

**Fig. 3:**
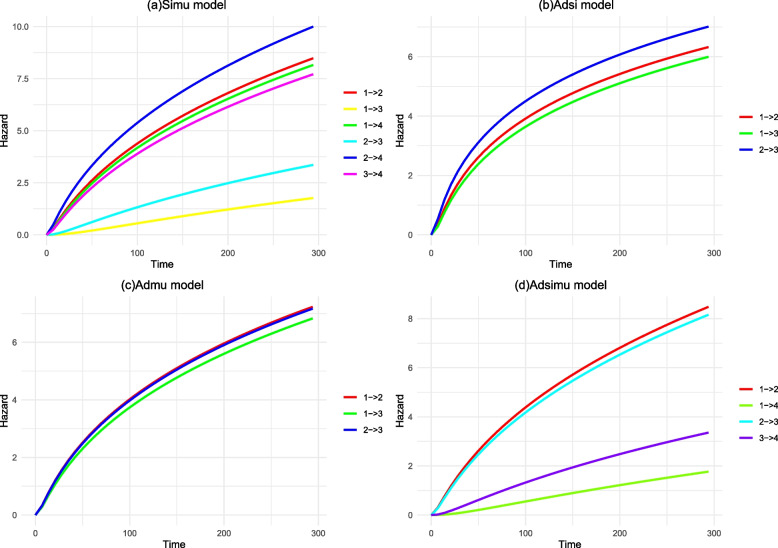
Transfer cumulative risk without stratification. The diagram (**a**) illustrates the cumulative risk curve of the mixed model, with state 1 indicating admission, state 2 representing single nosocomial infection, state 3 denoting multiple nosocomial infection, and state 4 signifying discharge. The diagram (**b**) illustrates the cumulative risk curve of the Adsi model, with state 1 indicating admission, state 2 representing single nosocomial infection, and state 3 denoting discharge. The Admu model's transfer-specific cumulative risk curve (**c**) depicts admission as state 1, multiple nosocomial infection as state 2, and discharge as state 3. The diagram (**d**) displays the cumulative risk curve of the Adsimu model, with state 1 representing admission, state 2 indicating single nosocomial infection, state 3 representing multiple nosocomial infection generations, and state 4 denoting discharge

The cumulative cause-specific hazards increased over time for each model. The mixed-model showed a higher cumulative hazard of infection at SNI rather than MNI. The cumulative risk of discharge with a SNI was found to be higher in the Adsi model compared to patients with a SNI. The cumulative risk of a patient having an infection at one or multiple sites in the Adsimu model was significantly higher than the risk of being discharged after infection. Refer to Fig. 3.

**Fig. 4:**
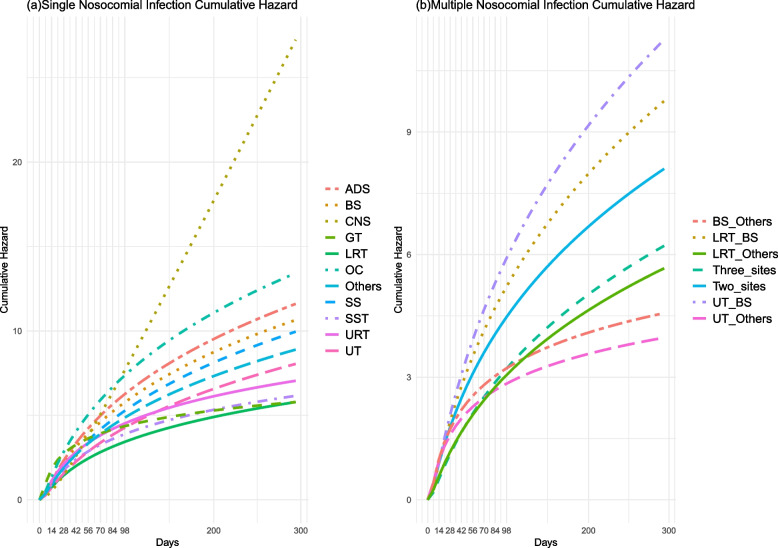
The cumulative risk curves stratified by infection sites of nosocomial infection. The diagram (**a**) represents cumulative risk of nosocomial infection at a single site, while the diagram (**b**) represents it at multiple sites. CNS; Lower Respiratory Tract, LRT; Surgical Site, SS; Blood System, BS; Urinary Tract, UT; Skin and Soft Tissue, SST; Abdomen and Digestive System, ADS; Upper Respiratory Tract, URT; Genital Tract, GT; Oral Cavity, OC

The cumulative risk curves in the SNI stratified model were found to be site-specific. The risk of GT infection peaked at 28 days and gradually declined to its lowest point after 266 days. The risk of OC infection was highest between days 28 and 91, surpassing that of GT infection. The risk of CNS infection was lowest within 28 days and highest after 91 days, surpassing that of OC infection. The risk of LRT infection reached its minimum after 28 days and remained consistently low (refer to Fig. 4a).

The risk of UT & BS infections increased after 21 days in MNI stratified models. The risk of infections at two sites was higher than that of infections at more than two sites, while LRT & others infections had a higher cumulative risk than UT & Others infections at 77 days. Refer to Fig. 4b.

### Prediction of transition specificity probabilities for MSMs

**Fig. 5:**
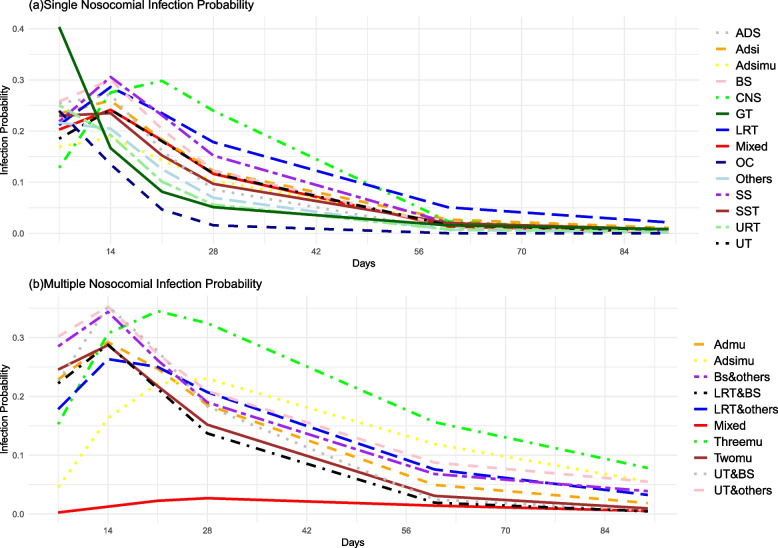
Predict nosocomial infection probability. The diagram(**a**) shows the probability of single nosocomial infection occurring at 7, 14, 21, 28, 60, and 90 days based on the infection site. The diagram(**b**) illustrates the probability of multiple nosocomial infection occurring at 7, 14, 21, 28, 60, and 90 days based on the infection site. Abdomen and Digestive System, ADS; Adsi model, Adsi; Adsimu model, Adsimu; Blood System, BS; Central Nervous System, CNS; Genital Tract, GT; Lower Respiratory Tract, LRT; Mixed model, Mixed; Oral Cavity, OC; Surgical Site, SS; Skin and Soft Tissue, SST; Upper Respiratory Tract, URT; Urinary Tract, UT; Admu model, Admu; Three sites of nosocomial infections, Threemu; Two sites of nosocomial infections, Twomu

In the SNI models, the probability of most SNI increased from day 7 to day 14, peaked at day 14, and gradually declined to its lowest point at day 90. The probability of GT infection peaked at 7 days and gradually decreased, while the probability of CNS infection reached its highest point at 21 days. In the MNI model, most models showed the highest probability of MNI at 14 days. However, the probability of more than two sites infection reached its peak at 21 days, and the transition from SNI to MNI was highest at 28 days. Refer to Fig. 5.

### Extra LOS due to NI

**Fig. 6:**
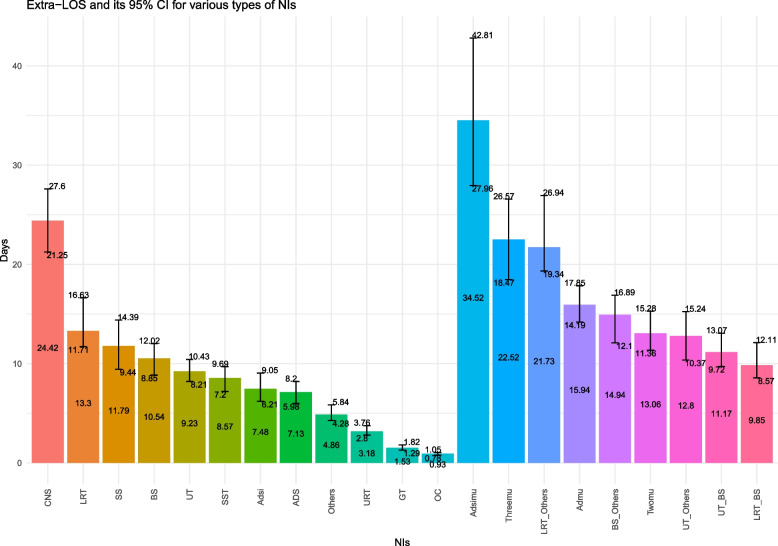
Extra − LOS and its 95% CI for various types of NIs. The figure showcases the varying distributions and patterns of extra-LOS among diverse NIs hospitalizations. The figure is composed of columns representing the extra-LOS for each hospitalization type. Of note, each column is accompanied by a line segment, representing the 95% CI for the corresponding extra-LOS value. Specifically, the upper endpoint of the line segment signifies the upper limit of the 95% CI (up95%CI), while the lower endpoint marks the lower limit (low95%CI). Note. Length of Stay, LOS; Confidence Interval, CI; Nosocomial Infection, NI; Adsi model, Adsi; Central Nervous System, CNS; Lower Respiratory Tract, LRT; Surgical Site, SS; Blood System, BS; Urinary Tract, UT; Skin and Soft Tissue, SST; Abdomen and Digestive System, ADS; Upper Respiratory Tract, URT; Genital Tract, GT; Oral Cavity, OC; Admu model, Admu; Three sites of nosocomial infections, Threemu; Two sites of nosocomial infections, Twomu; Adsimu model, Adsimu

The unstratified model showed that SNI increased LOS by 7.48 days (95%CI: 6.06–8.68). In the stratified model, CNS infections had the longest LOS at 24.42 days (95%CI: 20.76–29.55), followed by LRT infections with a prolonged LOS of 13.30 days (95%CI: 11.04–15.96). OC infection had the shortest impact on LOS, only extending it by 0.93 days (95%CI: 0.79–1.06). The LOS was prolonged by MNI in the MNI model, with a duration of 15.94 days (95%CI: 14.03–18.17) before stratification. After stratification, infections at more than two sites had the greatest impact on prolonging the LOS (22.52 days, 95%CI: 19.82–26.35), while LRT infections combined with BS had the least impact (9.85 days, 95%CI: 8.76–11.62). Notably, patients who experienced SNI followed by MNI had the longest LOS (34.52 days, 95%CI: 30.03–39.7). Please refer to Fig. 6.

## Discusscion

The Markov MSM, introduced by Soviet mathematician Markov between 1906 and 1912, is extensively applied in the study of epidemics and chronic diseases [28]. The Markov MSM operates under the assumption that future transitions depend solely on the current state, a principle known as the Markov property [29]. This implies that the model does not account for the aftereffect or the impact of previous states on future transitions. While this assumption simplifies the model, it may not accurately reflect the progression of some diseases, such as NIs, where the LOS can influence the risk of infection [30].

The semi-Markov MSM offers an enhancement over the traditional Markov MSM by incorporating the distribution of sojourn times in the current state before transitioning to the next [31]. This feature allows the semi-Markov MSM to capture the variability in the duration of state occupancy, which is particularly relevant for NI data. Unlike the Markov MSM, which often assumes an exponential distribution for state durations, the semi-Markov MSM can accommodate a range of distribution types, providing a more flexible and realistic model for analyzing NI data.

Patients with community-acquired infections were included in our study due to their susceptibility to NIs, even if they were infected upon admission. This was done to investigate the impact of being infected upon admission on patients suffering from NI. The PSM method was used to match patients with NIs and non-NIs, followed by MSM analysis on the matched dataset. Despite reducing the control group's sample size, the case group's sample size remained intact. After matching the dataset, baseline conditions of both groups were balanced, significantly shortening the operation time for MSM analysis using R software.

In the transfer specific regression, factors such as age, AI, male, surgery, CVC, and IC are promoting factors for the transfer of admission to SNI, which are common risk factors for NI [1, 32]. Age and AI are risk factors for the development of MNIs in patients with SNIs, while AI and surgery increase the risk of developing MNIs after admission. This finding suggests that elderly, surgical, and community-acquired infection patients are at a heightened risk for MNIs. Greater attention should be given to protecting this population. The rational and standardized use of antibiotics, especially in combination, should follow medication indications to avoid inducing bacterial resistance [33], which negatively impacts infection treatment. For instance, specific regression analysis revealed that the combination of antibiotics was identified as risk factor for the progression from SNI to MNI, which may be the result of non-standard medication.

The transfer-specific cumulative risk curve demonstrated a gradual increase in the risk of NI over time [34]. SNIs had a higher cumulative risk than MNIs, and two-site MNIs had a higher cumulative risk than more than two site MNIs. Discharge from the hospital with a SNI also carried a higher cumulative risk compared to MNI, indicating that while MNI posed lower risks, it was more challenging to treat. The cumulative risk curve, stratified by infection site, demonstrated the specific nature of each infection site's cumulative risk. This specificity in the cumulative risk of infection may have implications for personalized prevention and treatment of NIs.

The probability of infection does not increase linearly with LOS, but rather follows a wave pattern—initially rising and then falling [35]—due to the presence of competing events such as discharge and death [36] in addition to NIs. The study found that most NIs, whether single or multiple, are likely to occur within 14 days after admission, which aligns with the findings of Stewart S et al. [17]. The onset of GT infection is early, while CNS infection tends to be delayed. As the number of infection sites increases, the peak probability of occurrence is also delayed. Therefore, we should aim to shorten LOS, reduce unnecessary admissions, and minimize the risk of NIs.

The use of MSMs has several advantages over case–control studies alone in calculating the excess LOS due to NI. For instance, these models address time-dependent bias [37, 38], competing events influence [39], and avoid biases related to both time-dependent bias and competing risks [40]. Additionally, they consider the LOS before infection to prevent excessive results [41]. Therefore, the result of this study shows that NI has a smaller impact on the LOS compared to ordinary matched control studies [42]. The study found that SNI increased the LOS by 7.48 days, similar to the 7.8 days reported by Stewart S et al. [17] using a MSM. LRT infection extended the LOS by 16.3 days, BS infection prolonged it by 11.4 days, and SS infection added an extra 9.8 days to LOS, which is consistent with our findings. The result (8.09 ± 0.91 days) obtained by Habibollah et al. [2] using the MSM was similar to ours, and they also found that CNS infections had the longest LOS (21.28 ± 8.09 days), which is consistent with our finding of 24.42 days. The result of this study differs significantly from another national hospital in China (2.56 days) [3], possibly due to the higher grade and more severe condition of patients in that hospital, as well as longer LOS for the control group.

The study found that MNIs (unstratified) extended LOS by 15.94 days, similar to the findings of Kritsotakis et al. [18] (16.6 days). The greater the number of infection sites, the longer the extra LOS [43, 44], indicating that treatment becomes more challenging with an increasing number of infection sites. By calculating the extended LOS caused by NIs, it can be used to determine the direct economic losses associated with NIs. This has significant practical value in areas like cost accounting for prevention and control, evaluation of preventive measures' effectiveness, and medical insurance payment accounting.

The study has some limitations: for instance, the sample size of MNI is small, particularly in the stratified analysis where some layers have insufficient samples, resulting in low statistical power. In future research, we plan to extend the sampling time frame to enhance the sample size. The effect of the NI pathogens was not considered. In the future, we plan to conduct a specialized study on NI pathogens using MSM. The semi-Markov model only considers a time-homogeneous stochastic process with a constant transfer risk. However, in practice, there may still be situations where the risk is not constant, requiring further research. The study is multicenter, but it only uses data from one area, so the results may be specific to that region and differ from other studies.

## Conclusion

In conclusion, this study utilized a flexible parametric MSM based on cause-specific risk to simulate and analyze the occurrence and progression of SNIs and MNIs, aligning with their natural history. The risk, occurrence probability, and extra LOS are higher for certain SNIs like CNS infections and LRT infections. Although the cumulative risk and probability of MNI are lower than those of SNI, the extra LOS caused by MNI is longer. Targeted measures should be formulated and implemented to prevent and control key infection sites as well as MNIs.

### Supplementary Information

Supplementary Material 1.

## Data Availability

The data utilized in the study was obtained from the infection management department of Guangming District People Hospital. Please contact the author via email.

## Abbreviations

MNI: Multiple Nosocomial Infections
LOS: Length of Stay
MSM: Multi-State Model
SNI: Single Nosocomial Infection
ICU: Intensive Care Units
DRG: Diagnosis Related Group
RW: Relative Weight
PSM: Propensity score matching
LRT: Lower Respiratory Tract
SS: Surgical Site
BS: Blood System
SST: Skin and Soft Tissue
ADS: Abdomen and Digestive System
UT: Urinary Tract
URT: Upper Respiratory Tract
GT: Genital Tract
CNS: Central Nervous System
OC: Oral Cavity
CI: Confidence Intervals
SD: Standard Deviation
AI: Admission with Infection
IMV: Invasive Mechanical Ventilation
CVC: Central Venous Catheterization
IC: Indwelling Catheter
Use.Antibiotics: Use of Antibiotics
Coantibiotics: Combined Use of Antibiotics
HR: Hazard Ratio
