# Supplementary material for: Investigation of multiple nosocomial infections using a semi-Markov multi-state model
Source: Antimicrob Resist Infect Control. 2024 Jun 6;13:58. doi: 10.1186/s13756-024-01421-5 (PMC11157730; doi:10.1186/s13756-024-01421-5)
Supplement: Supplementary file 1 — Supplementary Material 1. [file 13756_2024_1421_MOESM1_ESM.docx]

**Diagnostic Criteria for Nosocomial Infections (Trial)**

**Definition of Nosocomial Infection**

Nosocomial infection refers to an infection acquired by a patient while hospitalized, including infections that occur during the hospital stay and those acquired in the hospital but manifest after discharge. It does not include infections that have begun or are present at the time of admission. Infections acquired by hospital staff within the hospital are also considered nosocomial infections.

Explanation:

**I. The following situations are considered nosocomial infections:**

1. For infections without a clear incubation period, those occurring more than 48 hours after admission are considered nosocomial infections; for infections with a clear incubation period, those occurring beyond the average incubation period starting from admission are considered nosocomial infections.

2. The current infection is directly related to the previous hospitalization.

3. New infections at other sites on the basis of the original infection (excluding metastatic foci of sepsis), or the isolation of new pathogens on the basis of known pathogens from the original infection (excluding contamination and original mixed infections).

4. Infections acquired by newborns during the birth process and postpartum.

5. Latent infections activated by diagnostic and therapeutic measures, such as infections with herpesviruses, Mycobacterium tuberculosis, etc.

6. Infections acquired by medical personnel during their work in the hospital.

**II. The following situations are not considered nosocomial infections:**

1. Open wounds of the skin and mucous membranes with bacterial colonization but without signs of inflammation.

2. Inflammatory manifestations caused by trauma or non-biological factors.

3. Infections acquired by newborns through the placenta (manifesting within 48 hours after birth), such as herpes simplex, toxoplasmosis, varicella, etc.

4. Acute exacerbations of chronic infections that the patient had prior to hospitalization.

Nosocomial infections should be reported based on clinical diagnosis, with efforts made to establish an etiological diagnosis.

**Respiratory System**

**I. Upper Respiratory Tract Infections**

Clinical Diagnosis

Fever (≥38.0^°^C for more than 2 days), with acute inflammatory manifestations of the nasopharynx, paranasal sinuses, and tonsils, etc.

Etiological Diagnosis

On the basis of clinical diagnosis, meaningful pathogenic microorganisms can be found in smears or cultures of secretions.

Explanation:

It is necessary to exclude acute inflammation of the upper respiratory tract caused by the common cold and non-infectious causes (such as allergies).

**II. Lower Respiratory Tract Infections**

Clinical Diagnosis

Diagnosis can be made if one of the following two conditions is met:

1. The patient has cough, viscous sputum, and moist rales in the lungs, and has one of the following:

(a) Fever.

(b) Increased total white blood cell count and (or) increased proportion of neutrophils.

(c) X-ray shows inflammatory infiltrative changes in the lungs.

2. Patients with chronic airway diseases in a stable phase (chronic bronchitis with or without obstructive emphysema, asthma, bronchiectasis) develop secondary acute infections, and there are etiological changes or obvious changes or new lesions in the chest X-ray compared to admission.

Etiological Diagnosis

On the basis of clinical diagnosis, diagnosis can be made if one of the following six conditions is met:

1. The same pathogen is isolated continuously from two sputum samples selected.

2. The number of pathogenic bacteria in sputum quantitative culture is ≥10^6^cfu/ml.

3. The pathogen is isolated from blood culture or pleural effusion in patients with complications.

4. The number of pathogenic bacteria in lower respiratory tract secretions collected by fiberoptic bronchoscopy or artificial airway aspiration is ≥10^5^cfu/ml; the number of pathogenic bacteria isolated by bronchoalveolar lavage (BAL) is ≥10^4^cfu/ml; or the number of pathogenic bacteria isolated from lower respiratory tract secretions collected by protected specimen brush (PSB), protected bronchoalveolar lavage (PBAL) must be ≥10^3^cfu/ml for patients with chronic obstructive pulmonary disease including bronchiectasis.

5. Bacteria that are not usually colonized in the respiratory tract or other special pathogens are isolated from sputum or lower respiratory tract samples.

6. Serological and pathological evidence of etiological diagnosis.

Explanation:

1. The standard for sputum screening is less than 10 squamous epithelial cells per low-power field and more than 25 white blood cells per low-power field, or a ratio of squamous epithelial cells to white blood cells ≤1:2.5; in immunosuppressed and neutrophil-deficient patients, the presence of columnar or conical epithelial cells and white blood cells can be not strictly limited.

2. Non-infectious causes such as pulmonary embolism, heart failure, pulmonary edema, lung cancer, etc., should be excluded as causes of changes in the chest X-ray of the lower respiratory tract.

3. Lesions limited to the airway are considered hospital-acquired tracheobronchitis; those with pulmonary parenchymal inflammation (as shown by X-ray) are considered hospital-acquired pneumonia (including lung abscess), and should be clearly marked when reported.

**III. Pleural Cavity Infections**

Clinical Diagnosis

Fever, chest pain, pleural effusion with purulent or foul-smelling appearance, and routine examination with white blood cell count ≥1000×10^^^6/L.

Etiological Diagnosis

On the basis of clinical diagnosis, diagnosis can be made if one of the following two conditions is met:

1. Pathogenic bacteria are isolated from pleural effusion culture.

2. No bacterial growth in routine culture of pleural effusion, but bacteria are seen in smears.

Explanation:

1. If pathogenic bacteria are found in pleural effusion, an etiological diagnosis can be made regardless of the nature of the pleural effusion and the results of routine examination.

2. Emphasis should be placed on the anaerobic culture of pleural effusion.

3. Pleural cavity infections naturally spreading from adjacent infections, such as those complicated by pneumonia, bronchopleural fistula, liver abscess, etc., are not considered nosocomial infections; infections caused by diagnostic procedures that promote the spread of infection are considered nosocomial infections. If pneumonia is a nosocomial infection, and its complication of empyema is reported according to nosocomial pneumonia, it should be marked with parentheses to indicate empyema.

4. Tuberculous pleurisy naturally evolving into tuberculous empyema is not considered a nosocomial infection.

5. When a patient has both upper and lower respiratory tract infections, only the lower respiratory tract infection needs to be reported.

**Cardiovascular System**

**I. Endocarditis involving cardiac valves (including prosthetic valves)**

Clinical Diagnosis

Patients must have at least two of the following symptoms or signs without another clear cause that can explain them: fever, new or changed heart murmurs, embolic phenomena, skin abnormalities (such as petechiae, bleeding, painful subcutaneous masses), congestive heart failure, cardiac conduction abnormalities, and combined with one of the following conditions:

1. Surgical procedures or pathological histology findings of cardiac vegetations.
2. Echocardiographic evidence of vegetations.

Etiological Diagnosis

On the basis of clinical diagnosis, one of the following three can be diagnosed:

1. Culturing of pathogens from cardiac valves or vegetations.
2. Two or more positive blood cultures on the basis of clinical diagnosis.
3. Gram staining of cardiac valves finding pathogens on the basis of clinical diagnosis.

**II. Myocarditis or Pericarditis**

Clinical Diagnosis

Diagnosis can be made if one of the following two is met.

1. Patients have at least two of the following symptoms or signs without another clear cause that can explain them: fever, chest pain, paradoxical pulse, cardiac enlargement, and combined with one of the following conditions:

(a) Abnormal electrocardiogram changes indicative of myocarditis or pericarditis.

(b) Evidence from cardiac tissue pathological examination.

(c) Imaging findings of pericardial effusion.

2. Patients aged 1 year or younger with at least two of the following symptoms or signs without another clear cause that can explain them: fever, chest pain, paradoxical pulse or cardiac enlargement, apnea, bradycardia, and at least one of the following conditions:

(a) Abnormal electrocardiogram changes indicative of myocarditis or pericarditis.

(b) Evidence from cardiac tissue pathological examination.

(c) Imaging findings of pericardial effusion.

Etiological Diagnosis

On the basis of clinical diagnosis, one of the following two can be diagnosed:

1. Culturing of pathogens from pericardial tissue or surgical/aspirated material.
2. Positive antibodies in the blood (such as Haemophilus influenzae, Streptococcus pneumoniae) on the basis of clinical diagnosis, excluding other site infections.

**Hematologic System**

**I. Vascular-Related Infections**

Clinical Diagnosis

Diagnosis can be made if one of the following three is met.

1. Purulent discharge from the venipuncture site, or the presence of diffuse erythema (a sign of cellulitis).
2. Painful diffuse erythema along the subcutaneous course of the catheter, excluding those caused by physicochemical factors.
3. Post-vascular interventional procedures, fever >38^°^C, local tenderness, with no other explainable causes.

Etiological Diagnosis

Culturing of meaningful pathogenic microorganisms from the catheter tip and/or blood culture.

Explanation:

1. The culture of the catheter tip should be taken from the 5 cm of the catheter tip, rolled back and forth once on the surface of a blood agar plate, and a bacterial count ≥15cfu/plate is considered positive.
2. Quantitative culture of blood drawn from the puncture site, with a bacterial count ≥100cfu/ml, or a bacterial count equivalent to 4-10 times that of simultaneous blood culture taken from the opposite side; or the same bacteria cultured from simultaneous blood culture taken from the opposite side.

**II. Sepsis**

Clinical Diagnosis

Fever >38^°^C or hypothermia <36^°^C, which may be accompanied by chills, and combined with one of the following conditions:

1. Presence of an entry portal or metastatic focus.
2. Systemic toxic symptoms without an obvious focus of infection.
3. Presence of petechiae or bleeding points, hepatosplenomegaly, and a significant increase in neutrophils with a shift to the left in the blood, with no other explainable causes.
4. Systolic blood pressure lower than 12kPa (90mmHg), or a decrease of more than 5.3kPa (40mmHg) from the original systolic blood pressure.

Etiological Diagnosis

On the basis of clinical diagnosis, one of the following two can be diagnosed:

1. Pathogenic microorganisms isolated from blood culture.
2. Detection of pathogen antigens in the blood.

Explanation:

1. Sepsis confirmed by blood culture at admission, and the appearance of new non-contaminated bacteria in blood culture after admission, or the appearance of new non-contaminated bacteria during the course of hospital sepsis, are all considered another episode of hospital-acquired septicemia.
2. Common skin bacteria isolated from blood culture, such as Corynebacterium, Enterobacteriaceae, coagulase-negative Staphylococci, Propionibacterium, etc., require blood to be drawn at different times, with two or more positive cultures.
3. Detection of pathogen antigens in the blood, such as Haemophilus influenzae, Streptococcus pneumoniae, Group B Streptococcus, must be consistent with symptoms and signs, and unrelated to other sites of infection.
4. Vascular-related sepsis (bacterial) belongs to this category, and catheter-related arteriovenous inflammation is included in cardiovascular infections.
5. Blood culture with multiple bacteria growth, after excluding contamination, can be considered multiple bacterial septicemia.

**III. Transfusion-Related Infections**

Commonly include viral hepatitis (B, C, D, E, etc.), AIDS, cytomegalovirus infection, malaria, toxoplasmosis, etc.

Clinical Diagnosis

Diagnosis can only be made if the following three conditions are met simultaneously.

1. The time from transfusion to onset of disease, or from transfusion to the appearance of pathogen immunological markers in the blood exceeds the average incubation period of that pathogen infection.
2. The recipient has never had this kind of infection before transfusion, and the immunological markers are negative.
3. Confirmation of the presence of infectious substances in the donor's blood, such as: detection of pathogens in the blood, positive immunological markers, positive pathogen DNA or RNA, etc.

Etiological Diagnosis

On the basis of clinical diagnosis, one of the following four can be diagnosed:

1. Pathogens found in the blood.
2. Positive detection of specific pathogen antigens in the blood, or the serum reaching a diagnostic level of IgM antibody titer, or a fourfold increase in IgG in two serum samples.
3. Inclusion bodies found in tissue or body fluid smears.
4. Confirmation by pathological biopsy.

Explanation:

1. Patients may have symptoms, signs, or only immunological changes.
2. The latency period of AIDS is long, and recipients may test positive for HIV antibodies within 6 months after transfusion, which can be used as a preliminary basis for diagnosis, but further confirmation tests are needed.

**Abdominal and Digestive System**

**I. Infectious Diarrhea**

Clinical Diagnosis

Diagnosis can be made if one of the following three is met.

1. Acute diarrhea, with white blood cells in the stool routine microscopy ≥10 per high-power field.
2. Acute diarrhea, or accompanied by fever, nausea, vomiting, abdominal pain, etc.
3. Acute diarrhea more than 3 times a day for 2 consecutive days, or more than 5 times of watery diarrhea in one day.

Etiological Diagnosis

On the basis of clinical diagnosis, diagnosis can be made if one of the following four is met.

1. Culturing of intestinal pathogens from stool or anal swab specimens.
2. Routine microscopy or direct electron microscopy examination of intestinal pathogens.
3. Detection of pathogen antigens or antibodies in blood or stool, reaching diagnostic criteria.
4. Determination of intestinal pathogens from pathological changes of cultured cells (such as toxin assay).

**Explanation:**

1. The number of acute diarrhea should be ≥3 times/24 hours.
2. Acute exacerbations of chronic diarrhea and diarrhea caused by non-infectious factors such as diagnostic and treatment reasons, underlying diseases, psychological tension, etc., should be excluded.

**II. Gastrointestinal Infections**

Clinical Diagnosis

Patients exhibit fever (≥38°C), nausea, vomiting, and (or) abdominal pain, diarrhea, with no other cause that can explain.

Etiological Diagnosis

On the basis of clinical diagnosis, diagnosis can be made if one of the following three is met.

1. Culturing of pathogens from tissue specimens obtained by surgical operation or endoscopy, or from surgical drainage fluid.
2. Pathogens or multinucleated giant cells visible by Gram staining or potassium hydroxide floatation slide of the above specimens.
3. Surgical or endoscopic specimens show pathological evidence of infection.

**III. Antibiotic-Associated Diarrhea**

Clinical Diagnosis

Recently used or currently using antibiotics, with the occurrence of diarrhea, which may be accompanied by changes in stool characteristics such as watery stool, bloody stool, mucopurulent stool, or pseudomembrane with patchy or ribbon-like appearance, and may be combined with one of the following conditions:

1. Fever ≥38^°^C.
2. Abdominal pain or abdominal tenderness, rebound tenderness.
3. Leukocytosis in peripheral blood.

Etiological Diagnosis

On the basis of clinical diagnosis, diagnosis can be made if one of the following three is met.

1. Stool smear showing dysbiosis of the flora or culture revealing a significant predominance of certain bacterial groups.

2. If feasible, a fiberoptic colonoscopy reveals signs of intestinal wall congestion, edema, bleeding, or the presence of gray-yellow (white) patchy pseudomembranes ranging from 2mm to 20mm in size.

3. Confirmation through bacterial toxin assays.

Explanation:

1. The number of acute diarrhea episodes should be ≥3 times/24 hours.

2. Acute flare-ups of chronic enteritis or acute gastrointestinal infections and diarrhea caused by non-infectious reasons should be excluded.

**IV. Viral Hepatitis**

Clinical Diagnosis

A history of blood transfusion or use of blood products, consumption of unclean food, or contact with hepatitis, with any two of the following symptoms or signs and abnormal liver function, without any other explainable causes.

1. Fever.

2. Anorexia.

3. Nausea and vomiting.

4. Pain in the hepatic region.

5. Jaundice.

Etiological Diagnosis

On the basis of clinical diagnosis, positivity for any serological markers of active viral hepatitis A, B, C, D, E, or other types.

Explanation:

Non-infectious causes (such as α1-antitrypsin deficiency, alcohol, drugs, etc.) and hepatitis or damage caused by biliary tract diseases should be excluded.

**V. Intra-abdominal (Pelvic) Tissue Infections**

Includes acute infections of the gallbladder, bile ducts, liver, spleen, pancreas, peritoneum, subphrenic spaces, pelvis, and other tissues or cavities, including secondary peritonitis due to continuous peritoneal dialysis.

Clinical Diagnosis

Presence of any two of the following symptoms or signs without any other explainable cause, along with corresponding abnormalities found in laboratory tests or imaging studies.

1. Fever ≥38°C.

2. Nausea and vomiting.

3. Abdominal pain, tenderness, rebound tenderness, or palpable mass with tenderness.

4. Jaundice.

Etiological Diagnosis

On the basis of clinical diagnosis, diagnosis can be made if one of the following two is met.

1. Pathogens detected in specimens obtained through surgery, drainage tubes, aspiration, or endoscopy.

2. Positive blood culture, consistent with the local infection bacteria or in line with clinical presentation.

Explanation:

1. Inflammatory responses caused by non-biological factors and acute exacerbations of chronic infections should be excluded.

2. Infections caused by primary organ perforation are not considered nosocomial infections.

**VI. Ascites Infection**

Clinical Diagnosis

Ascites that was originally an exudate, with the presence of either of the following can be diagnosed.

1. Ascites examination changes to an exudative fluid.

2. Ascites is not easily eliminated, with abdominal pain, tenderness, or rebound tenderness. Routine ascites examination with white blood cells >200×10^^^6/L, neutrophils >25%.

Etiological Diagnosis

On the basis of clinical diagnosis, positive bacterial culture of ascites.

**Central Nervous System**

**I. Bacterial Meningitis, Ventriculitis**

Clinical Diagnosis

Diagnosis can be made if one of the following three is met.

1. Fever, one of the symptoms of increased intracranial pressure (headache, vomiting, high tension of infant's anterior fontanel, disturbance of consciousness), one of the signs of meningeal irritation (neck resistance, Brudzinski's sign, Kernig's sign, opisthotonos), inflammatory changes in cerebrospinal fluid (CSF).

2. Fever, symptoms of increased intracranial pressure, meningeal irritation, and mild to moderate increase in white blood cells in CSF, or after antimicrobial drug treatment, the symptoms and signs disappear, and CSF returns to normal.

3. During the use of antibiotics, fever, atypical symptoms and signs of increased intracranial pressure, mild increase in white blood cells in CSF, and one of the following situations:

(a) Anti-specific pathogen IgM in CSF reaches the diagnostic standard, or IgG increases fourfold, or bacteria are found in CSF smear.

(b) History of invasive operations on the skull and brain (such as skull and brain surgery, intracranial puncture, intracranial implants), or history of skull and brain trauma or lumbar puncture.

(c) Infection foci near the meninges (such as scalp incision infection, skull osteomyelitis, etc.) or presence of CSF leakage.

(d) Positive blood culture in newborns.

Etiological Diagnosis

On the basis of clinical diagnosis, diagnosis can be made if one of the following three is met.

1. Culturing of pathogenic bacteria in CSF.

2. Positive immunological test for pathogenic microorganisms in CSF.

3. Finding of pathogenic bacteria in CSF smear.

Explanation:

1. Infants under one year old with fever (>38^°^C) or hypothermia (<36^°^C), presenting with disturbance of consciousness, apnea, or convulsions, if no other cause can explain, should be suspected of meningitis and undergo relevant examinations in a timely manner.

2. Elderly people with low reactivity may only exhibit somnolence, reduced conscious activity, and disorientation, and should undergo relevant examinations in a timely manner.

3. The key points of distinction between bacterial meningitis and traumatic meningitis, meningeal reaction of brain tumors are the reduction of CSF sugar, increased C-reactive protein, etc.

**II. Intracranial Abscess (including brain abscess, subdural and extradural abscesses, etc.)**

Clinical Diagnosis

Diagnosis can be made if one of the following two is met.

1. Fever, one of the symptoms of increased intracranial pressure, intracranial space-occupying signs (functional area localization signs), and one of the following imaging evidence:

(a) CT scan.

(b) Cerebral angiography.

(c) Magnetic resonance imaging.

(d) Radionuclide scanning.

2. Confirmed by surgical operation.

Etiological Diagnosis

On the basis of clinical diagnosis, the pathogen is found in the pus or tissue biopsy, or positive bacterial culture.

**III. Infections within the Spinal Canal**

Including subdural and intramedullary abscesses of the spinal cord.

Clinical Diagnosis

Diagnosis can be made if one of the following two is met.

1. Fever, neurological localization symptoms and signs or localized back pain and limited spinal movement, and one of the following situations:

(a) Severe tenderness and percussion pain around the spinous process.

(b) Radicular pain.

(c) Complete or incomplete signs of spinal cord compression.

(d) Examination confirmed: spinal CT, myelography, magnetic resonance imaging, X-ray plain film, increased protein and white blood cells in CSF and Queckenstedt's test showing partial or complete spinal blockage.

2. Confirmed by surgery.

Etiological Diagnosis

Positive bacterial culture of surgical drainage fluid.

Explanation:

1. Infections within the spinal canal complicated by meningitis are included in the statistical report of bacterial meningitis.

2. Such nosocomial infections are rare and mostly occur in patients with sepsis, inflammation near the spine, spinal trauma, or a history of high spinal anesthesia during surgery.

3. Metastatic foci of sepsis or spread of inflammation from the spine and adjacent areas should be excluded.

**Urinary System**

Clinical Diagnosis

Patients exhibit urinary tract irritation symptoms such as increased frequency of urination, urgency, and pain, or have lower abdominal tenderness, renal area percussion pain, with or without fever, and have one of the following:

1. Urine test with white blood cells in males ≥5 per high-power field, in females ≥10 per high-power field; patients with catheters should be combined with urine culture.

2. Clinically diagnosed with urinary tract infection, or urinary tract infection identified as effective with antimicrobial treatment.

Etiological Diagnosis

On the basis of clinical diagnosis, one of the following four can be diagnosed:

1. Clean midstream urine or urine collected by catheter (non-indwelling catheter) culture with Gram-positive cocci count ≥10^^^4 CFU/ml, Gram-negative bacilli count ≥10^^^5 CFU/ml.

2. Suprapubic bladder puncture to collect urine culture with bacterial count ≥10^^^3 CFU/ml.

3. Fresh urine specimen centrifuged and examined with phase-contrast microscopy (×400), with bacteria seen in half of the fields in 30 fields.

4. Asymptomatic bacteriuria: Although patients are asymptomatic, if they have a history of endoscopy or indwelling catheter in the recent period (usually 1 week), urine culture with Gram-positive cocci concentration ≥10^^^4 CFU/ml, Gram-negative bacilli concentration ≥10^^^5 CFU/ml should be considered as urinary system infection.

Explanation:

1. Bacterial culture results of non-catheter or puncture urine specimens with two or more types of bacteria should consider the possibility of contamination and suggest re-collection and examination of specimens.

2. Urine specimens should be inoculated in a timely manner. If urine specimens are placed at room temperature for more than 2 hours, even if the inoculated culture results show bacterial counts ≥10^^^4 or 10^^^5 CFU/ml, they should not be used as a basis for diagnosis and specimens should be recollected.

3. Infections of the urinary system (such as kidneys, perirenal tissues, ureters, bladder, urethra) confirmed by imaging, surgery, histopathology, or other methods should be clearly marked in the report.

**Surgical Site Infections**

**I. Superficial Surgical Site Infections**

Limited to the skin and subcutaneous tissues involved by the incision, occurring within 30 days postoperatively.

Clinical Diagnosis

Diagnosis can be made with one of the following:

1. Redness, swelling, heat, pain, or purulent discharge at the superficial incision site.

2. Superficial incision infection diagnosed by a clinician.

Microbiological Diagnosis

Positive bacterial culture based on clinical diagnosis.

Notes:

1. Wounds include surgical incisions and wounds caused by accidental injuries. To avoid confusion, the term "wound infection" is not used. Infections related to wounds are referred to the diagnostic criteria for skin and soft tissue infections.

2. Mild inflammation and a small amount of discharge at the suture site of the incision do not constitute an incision infection.

3. Fat liquefaction of the incision, with clear liquid, does not constitute an incision infection.

**II. Deep Surgical Site Infections**

Infections related to surgery occurring within 30 days postoperatively without implants, or within one year postoperatively with implants (such as prosthetic heart valves, artificial blood vessels, mechanical hearts, prosthetic joints, etc.), involving deep soft tissues of the incision (deep fascia and muscles).

Clinical Diagnosis

In accordance with the above provisions, and with one of the following, a diagnosis can be made:

1. Purulent discharge drained or aspirated from the deep incision, excluding infectious postoperative drainage fluid.

2. The incision naturally dehisces or is opened by a surgeon, with purulent discharge or fever ≥38°C, accompanied by local pain or tenderness.

3. Reoperation exploration, histopathological or imaging examination reveals evidence of deep incision abscess or other signs of infection.

4. Deep incision infection diagnosed by a clinician.

Microbiological Diagnosis

Positive bacterial culture from secretions based on clinical diagnosis.

**III. Organ (or Cavity) Infections**

Infections of organs or cavities related to surgery occurring within 30 days postoperatively without implants, or within one year postoperatively with implants (excluding skin, subcutaneous tissue, deep fascia, and muscles).

Clinical Diagnosis

In accordance with the above provisions, and with one of the following, a diagnosis can be made:

1. Purulent discharge from drainage or aspiration.

2. Reoperation exploration, histopathological or imaging examination reveals evidence of infection in organs (or cavities).

3. Infection of organs (or cavities) diagnosed by a clinician.

Microbiological Diagnosis

Positive bacterial culture based on clinical diagnosis.

Notes:

1. Typical surgical site infections shown by clinical and (or) relevant examinations can be diagnosed even if bacterial culture is negative.

2. When both superficial and deep parts of the surgical incision are infected, only the deep infection needs to be reported.

3. Infections of organs (or cavities) caused by incision drainage that do not require reoperation should be considered as deep incision infections.

**Skin and Soft Tissues**

**I. Skin Infections**

Clinical Diagnosis

Diagnosis can be made with one of the following:

1. Purulent discharge, pustules, boils, or abscesses on the skin.

2. Local pain or tenderness, local redness or warmth in the patient, without other reasons for explanation.

Microbiological Diagnosis

Based on clinical diagnosis, diagnosis can be made with one of the following:

1. Culturing of pathogens from drainage or aspiration of the infected site.

2. Positive detection of specific pathogen antigens in blood or infected tissues.

**II. Soft Tissue Infections**

Soft tissue infections include necrotizing fasciitis, infectious gangrene, necrotizing cellulitis, infectious myositis, lymphadenitis, and lymphangitis.

Clinical Diagnosis

Diagnosis can be made with one of the following:

1. Purulent discharge drained from the infected site.

2. Infection confirmed by surgical operation or tissue pathology examination.

3. Local pain or tenderness, local redness or warmth in the patient, without other reasons for explanation.

Microbiological Diagnosis

Based on clinical diagnosis, diagnosis can be made with one of the following:

1. Positive detection of specific pathogen antigens in blood, or diagnostic levels of serum IgM antibody titers, or a fourfold increase in paired serum IgG.

2. Culturing of pathogens from drainage or tissues of the infected site.

**III. Pressure Ulcer Infections**

Pressure ulcer infections include superficial and deep tissue infections of pressure ulcers.

Clinical Diagnosis

Local redness, tenderness, or swelling at the edge of the pressure ulcer, with purulent discharge.

Microbiological Diagnosis

Positive culture of secretions based on clinical diagnosis.

**IV. Burn Infections**

Clinical Diagnosis

Changes in the morphology or characteristics of the burn surface, such as rapid separation of eschar, eschar turning brown-black, black, or violet, edema at the edges of the burn. With one of the following:

1. Purulent discharge at the wound site.

2. The patient has a fever >38^°^C or hypothermia <36^°^C, combined with hypotension.

Microbiological Diagnosis

Based on clinical diagnosis, diagnosis can be made with one of the following:

1. Positive blood culture and exclusion of other site infections.

2. Biopsy of burned tissue showing microbial infiltration into adjacent tissues.

Notes:

1. Isolated fever cannot be diagnosed as burn infection because fever may be the result of tissue damage or the patient may have infections in other areas.

2. Graft rejection accompanied by clinical evidence of infection (inflammation or pus) is considered a hospital infection.

3. Donor site infections are considered burn infections.

**V. Breast Abscess or Mastitis**

Clinical Diagnosis

Diagnosis can be made with one of the following:

1. Signs of inflammation such as redness, swelling, heat, pain, or fever, excluding milk stasis in nursing women.

2. Confirmed by surgical operation.

3. Clinical diagnosis of breast abscess by a physician.

Microbiological Diagnosis

Positive culture of drainage or needle aspirate based on clinical diagnosis.

**VI. Omphalitis**

Clinical Diagnosis

Redness or purulent discharge at the umbilical site of a newborn.

Microbiological Diagnosis

Based on clinical diagnosis, diagnosis can be made with one of the following:

1. Positive culture of drainage or needle aspirate fluid.

2. Positive blood culture, excluding infections in other sites.

Note: Umbilical artery and vein infections related to umbilical catheterization should be classified as cardiovascular system infections.

**VII. Infantile Pyoderma**

Clinical Diagnosis

Diagnosis can be made with one of the following:

1. Appearance of pustules on the skin.

2. Clinical diagnosis of pyoderma by a physician.

Microbiological Diagnosis

Positive culture of secretions based on clinical diagnosis.

**Bones and Joints**

**I. Infections of Joints and Bursae**

Clinical Diagnosis

Diagnosis can be made with one of the following:

1. The patient has two of the following symptoms or signs without another reason that can explain: joint pain, swelling, tenderness, fever, effusion, or limited motion. Combined with one of the following situations:

(a) White blood cells found in joint fluid tests.

(b) The cellular composition and chemical examination of joint fluid are consistent with infection and cannot be explained by rheumatic diseases.

(c) Imaging evidence of infection.

2. Surgical operation or histopathological examination reveals evidence of infection in joints or bursae.

Microbiological Diagnosis

Diagnosis can be made with one of the following:

1. Culturing of pathogens from joint fluid or bursal biopsy.

2. Identification of pathogens by Gram staining of joint fluid on the basis of clinical diagnosis.

**II. Osteomyelitis**

Clinical Diagnosis

A diagnosis can be made if one of the following two conditions is met:

1. The patient exhibits two of the following symptoms or signs without any other explainable causes: fever (>38^°^C), local mass, tenderness, warmth, or purulent discharge from the site of infection, along with radiological evidence of infection.

2. Confirmation through surgical operation or histopathological examination.

Microbiological Diagnosis

A diagnosis can be made if one of the following two conditions is met:

1. Culturing of the pathogen from the bone marrow.

2. On the basis of clinical diagnosis, culturing of the pathogen from the blood or detection of bacterial antibodies in the blood (such as Haemophilus influenzae, Streptococcus pneumoniae), excluding infections in other sites.

**III. Intervertebral Disc Infection**

Clinical Diagnosis

A diagnosis can be made if one of the following three conditions is met:

1. The patient has unexplained fever or intervertebral disc pain, along with radiological evidence of infection.

2. Surgical operation or histopathological examination reveals evidence of intervertebral disc infection.

3. The intervertebral disc tissue, either surgically excised or aspirated by needle, confirms the presence of infection.

Microbiological Diagnosis

On the basis of clinical diagnosis, a diagnosis can be made if one of the following two conditions is met:

1. Culturing of the pathogen from the infected tissue.

2. Detection of antibodies in blood or urine (such as Haemophilus influenzae, Streptococcus pneumoniae, Neisseria meningitidis, or Group B Streptococcus), excluding infections in other sites.

**Reproductive Tract**

**I. Perineal Incision Infection**

Infection of the perineal incision occurring within 2 weeks postpartum after vaginal delivery.

Clinical Diagnosis

Diagnosis can be made in accordance with the above provisions and with one of the following two:

1. The perineal incision exhibits redness, swelling, heat, pain, or purulent discharge.

2. Abscess formation at the perineal incision.

Microbiological Diagnosis

Positive bacterial culture based on clinical diagnosis.

Notes:

1. Perineal incision infection includes episiotomy or sutured perineal laceration.

2. Mild inflammation and a small amount of discharge at the suture site do not constitute a perineal incision infection.

**II. Infection of the Vaginal Vault**

Clinical Diagnosis

Diagnosis can be made with one of the following:

1. Purulent discharge from the vaginal stump after hysterectomy.

2. Abscess formation at the vaginal stump after hysterectomy.

Microbiological Diagnosis

Positive bacterial culture based on clinical diagnosis.

Note:

Infection of the vaginal vault refers only to the area at the vaginal stump after total hysterectomy.

**III. Acute Pelvic Inflammatory Disease**

Clinical Diagnosis

Diagnosis can be made with one of the following:

1. Presence of the following symptoms or signs without another reason to explain: fever, nausea, vomiting, lower abdominal pain or tenderness, increased urinary frequency, urgency, or diarrhea, tenesmus, and increased purulent vaginal discharge.

2. Pus obtained from posterior fornix or abdominal puncture.

Microbiological Diagnosis

Positive bacterial culture of cervical canal secretions based on clinical diagnosis.

Note:

Limited to cases occurring after 48 hours of hospital admission, or after intrauterine invasive procedures, or within one week after natural childbirth within 24 hours of discharge.

**IV. Endometritis**

Clinical Diagnosis

Fever or chills, lower abdominal pain or tenderness, irregular vaginal bleeding, or foul-smelling lochia.

Microbiological Diagnosis

Confirmation by pathological examination of endometrial scrapings from the uterine cavity or positive bacterial culture of secretions based on clinical diagnosis.

Notes:

1. At admission, the patient has no amniotic fluid infection, and the rupture of membranes does not exceed 48 hours.

2. Endometritis includes only early pregnancy miscarriage, mid-pregnancy induced abortion, and within one week after childbirth.

**V. Other Infections of the Male and Female Reproductive Tracts**

Clinical Diagnosis

Diagnosis can be made with one of the following:

1. The patient has two of the following symptoms or signs without another reason to explain: fever, local pain, tenderness, or dysuria, with imaging or pathological confirmation.

2. Surgical operation or histopathological findings of abscesses or other evidence of infection at the infection site.

Microbiological Diagnosis

Diagnosis can be made with one of the following:

1. Culturing of pathogens from tissues or secretions at the infection site.

2. Culturing of pathogens in the blood based on clinical diagnosis.

**Oral Cavity**

Clinical Diagnosis

Diagnosis can be made with one of the following three:

1. Purulent discharge in oral tissues.

2. Oral infection or abscess confirmed by surgical operation or histopathological examination.

3. Infection diagnosed by a clinical physician and treated with oral antifungal therapy.

Microbiological Diagnosis

Based on clinical diagnosis, diagnosis can be made with one of the following five:

1. Identification of pathogenic microorganisms by Gram staining.

2. Positive potassium hydroxide staining.

3. Microscopic examination of mucosal scrapings showing multinucleated giant cells.

4. Positive antigen detection in oral secretions.

5. Diagnostic levels of IgM antibody titers or a fourfold increase in paired serum IgG.

Note:

Primary herpetic infections should be categorized under this type of infection.

**Other Sites**

Infections involving multiple organs or systems that do not fit into a specific system are usually viral infections, such as measles, rubella, infectious mononucleosis; viral exanthems should also be included in this category, such as herpes simplex, varicella, zoster, etc.
